# Peptide set test: a peptide-centric strategy to infer differentially expressed proteins

**DOI:** 10.1093/bioinformatics/btae270

**Published:** 2024-04-17

**Authors:** Junmin Wang, Steven Novick

**Affiliations:** Data Sciences and Quantitative Biology, Discovery Sciences, Biopharmaceuticals R&D, AstraZeneca, Gaithersburg, MD 20878, United States; Global Statistical Sciences, Eli Lilly, Indianapolis, IN 46285, United States

## Abstract

**Motivation:**

The clinical translation of mass spectrometry-based proteomics has been challenging due to limited statistical power caused by large technical variability and inter-patient heterogeneity. Bottom-up proteomics provides an indirect measurement of proteins through digested peptides. This raises the question whether peptide measurements can be used directly to better distinguish differentially expressed proteins.

**Results:**

We present a novel method called the peptide set test, which detects coordinated changes in the expression of peptides originating from the same protein and compares them to the rest of the peptidome. Applying our method to data from a published spike-in experiment and simulations demonstrates improved sensitivity without compromising precision, compared to aggregation-based approaches. Additionally, applying the peptide set test to compare the tumor proteomes of tamoxifen-sensitive and tamoxifen-resistant breast cancer patients reveals significant alterations in peptide levels of collagen XII, suggesting an association between collagen XII-mediated matrix reassembly and tamoxifen resistance. Our study establishes the peptide set test as a powerful peptide-centric strategy to infer differential expression in proteomics studies.

**Availability and implementation:**

Peptide set test (PepSetTest) is publicly available at https://github.com/JmWangBio/PepSetTest.

## 1 Introduction

Liquid chromatography-mass spectrometry (LC-MS/MS)-based proteomics has proven to be a sensitive and unbiased tool for accelerating the understanding of various diseases, including cancer(Suh *et al.* 2015, Shu *et al.* 2020, Chaerkady *et al.* 2021, Ding *et al.* 2022, Hristova *et al.* 2023). It enables the unraveling of disease mechanisms by revealing alterations in protein expression. Common study designs involve comparing two groups to determine, for example, whether the expression of a protein differs between two disease phenotypes.

In bottom-up proteomics, peptide abundance measured via LC-MS/MS is traditionally collapsed into protein abundance, and statistical analysis is then performed on the aggregated protein abundance to infer differentially expressed proteins (Nesvizhskii *et al.* 2003, Choi *et al.* 2014, Tyanova *et al.* 2016). The empirical Bayes moderated *t*-test implemented in the limma R package is among the most commonly used techniques for this purpose (Ritchie *et al.* 2015, Nuber *et al.* 2021, Chakrabarti *et al.* 2022). By leveraging the parallel nature of -omics data, this method borrows information across proteins to improve the reliability of conclusions drawn from statistics (Ritchie *et al.* 2015). However, despite its widespread use, the empirical Bayes method may still lack the required sensitivity. Technical variability in sample preparation, protein digestion, and instrumental stability can all contribute to the challenges faced by the LC-MS/MS platform, thereby affecting the statistical power of the study (Piehowski *et al.* 2013). Moreover, large inter-subject heterogeneity or subtle yet significant changes within compared cohorts pose additional complications, particularly in clinical settings (Suh *et al.* 2015, Chakrabarti *et al.* 2022, Hristova *et al.* 2023). These inherent statistical challenges drive the need for novel computational methods capable of extracting maximum insights from each dataset (Chandramouli and Qian 2009, Zhou *et al.* 2012).

In this study, we developed a peptide-centric approach by fitting a linear model to the peptide data followed by a novel competitive peptide set test. The peptide set test was designed to detect coordinated changes in the expression of peptides originating from a protein of interest (POI) and compare the magnitude of these changes against the rest of the peptidome, similar to competitive gene set tests (Wu and Smyth 2012). Our method can also account for inter-peptide correlation when evaluating differential expression. While our method was adapted from CaMERA, which stands for Correlation Adjusted Mean Rank gene set test (Wu and Smyth 2012), we introduced several important innovations to customize the statistical framework for proteomics data analysis. First, we utilized a mixed model to estimate inter-peptide correlation coefficients based on variance components. This approach assumed that peptides originating from the same protein are generally positively correlated, if such correlation exists, and that all peptides belonging to the same protein have equal pairwise correlation. Second, we replaced the sample standard deviation (SD) with the sample median absolute deviation (MAD) as a more robust estimator of the standard deviation in peptides not belonging to the POI.

By utilizing simulated data, we demonstrated that the competitive peptide set test achieved superior statistical power compared to the self-contained peptide set test and traditional aggregation-based approaches while correctly controlling the Type I error rate in nearly all cases. Similar to CaMERA, we adapted FRY, a self-contained gene set test, for the analysis of differential protein expression (Wu *et al.* 2010). While the competitive approach compares a peptide set against the background, a self-contained test determines whether a peptide set is differentially expressed with no reference to other peptides (see Subsection 2.4 for details) (Wu *et al.* 2010, Wu and Smyth 2012). To validate the effectiveness of our approach, we applied the competitive peptide set test to a published spike-in experiment (Paulovich *et al.* 2010). The results showed vastly improved sensitivity when compared to aggregation-based approaches. Furthermore, to illustrate its ability to recover biologically meaningful results, we also applied the competitive peptide set test to a published proteomics dataset to compare the tumor proteomes of tamoxifen-sensitive and tamoxifen-resistant estrogen receptor (ER)-positive breast cancer patients (De Marchi *et al.* 2016). Our analysis revealed significantly altered peptide levels of COL12A1 in tamoxifen-resistant patients, suggesting an association between collagen XII-mediated matrix reassembly and tamoxifen resistance in breast cancer.

## 2 Materials and methods

We aimed to develop a statistical test with improved sensitivity by following the principles of competitive gene set tests. A competitive gene set test infers the biological significance of gene signatures by comparing genes in the test set relative to all other genes (Wu and Smyth 2012). Similarly, peptides can be mapped to proteins, and based on this analogy, we developed a competitive peptide set test to infer differentially expressed proteins by directly utilizing peptide data rather than protein data.

### 2.1 Steps of the competitive peptide set test

**Figure 1.**
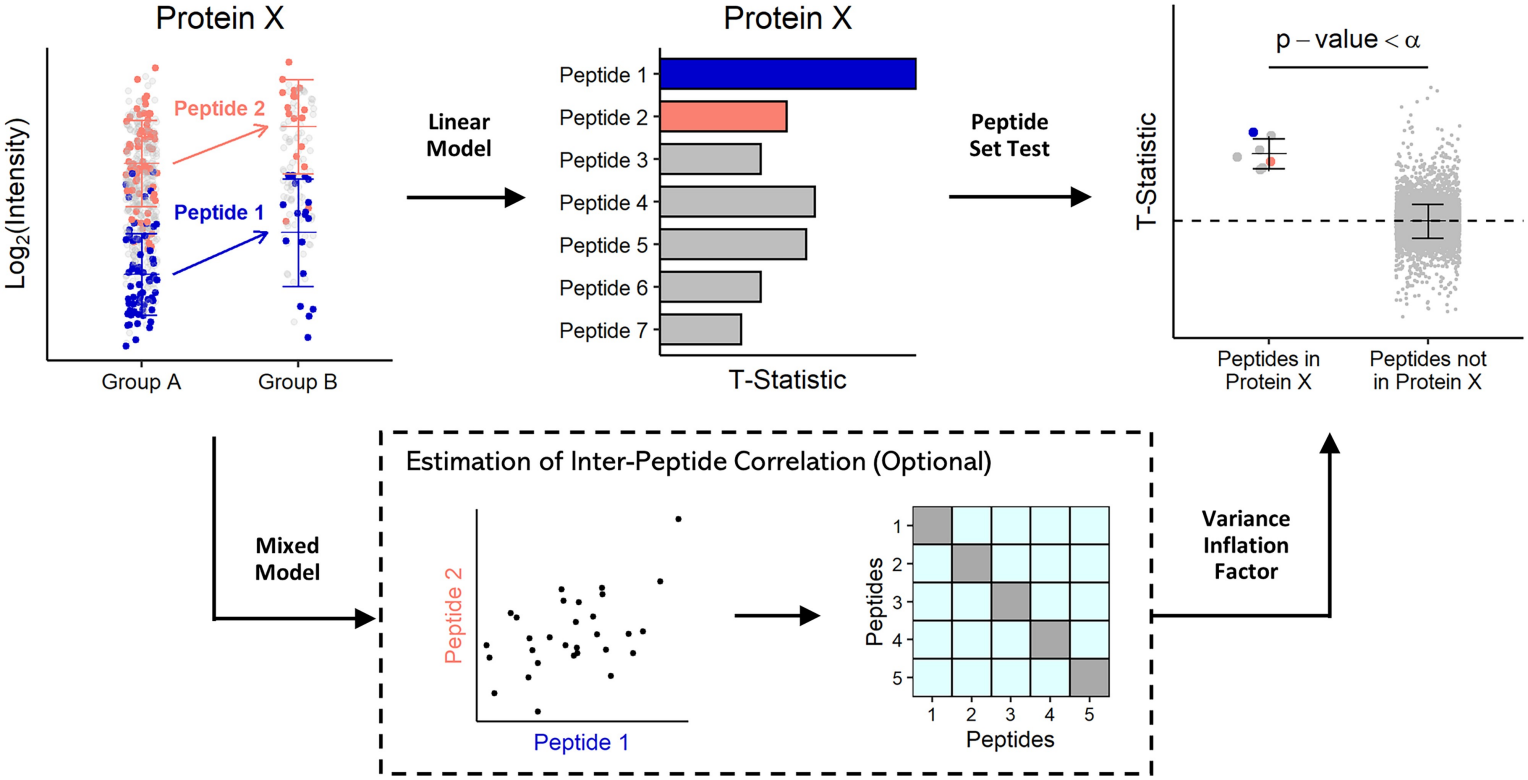
Diagram of the competitive peptide set test. The left jitter plot displays the abundance of all peptides mapped to protein X, with each dot representing a peptide. In the middle bar plot, the *t*-statistics of all peptides mapped to protein X are shown. The right jitter plot displays the *t*-statistics of peptides mapped to protein X compared to the rest of the peptidome. The error bars indicate mean ± pooled standard deviation. The variance of peptides not belonging to protein X can be estimated using either the sample SD or the sample MAD scaled by 1.4826. The inter-peptide correlation can be calculated using a mixed model approach. The *P*-value is calculated using a two-sample *t*-test that takes inter-peptide correlation into consideration.

The procedure of the competitive peptide set test is as follows. First, we divide the global peptidome into two sets: peptides that belong to the POI and peptides that do not belong to the POI (Fig. 1). Peptides belonging to the POI include both unique peptides and shared peptides mapped to the POI. Let P represent the indices of peptides belonging to the POI, and $P^{c}$ represent the complementary set of indices of peptides not belonging to this protein. We state the null hypothesis as: $\mu_{t_{P}}=\mu_{t_{P^{c}}}$. In other words, the average *t*-statistic of peptides from P is equal to that from $P^{c}$. This means that, under the null hypothesis, peptides belonging to the POI are no more differentially expressed than the rest of the peptides in the peptidome. The alternative hypothesis is: $\mu_{t_{P}}\neq \mu_{t_{P^{c}}}$.

Peptide-wise *t*-statistics are calculated by fitting a linear model to the peptide data (Fig. 1). The subsequent steps of the test depend on whether peptides originating from the same proteins are assumed to be correlated (Fig. 1). If they are non-correlated, the average *t*-statistics of P and $P^{c}$ are compared using an ordinary two-sided two-sample *t*-test. If they are correlated, a two-sided two-sample *t*-test accounting for inter-peptide correlation, as described in a previous study (Wu and Smyth 2012), is conducted.

Two options are available to estimate the inter-peptide correlation coefficient. If all peptides of the same protein are assumed to have equal pairwise correlation, a linear mixed model is fitted to the peptide data. The correlation coefficient is then calculated based on the estimated variance components (see Subsection 2.2 for details). This approach is referred to as the mixed model approach from here onwards. Otherwise, the correlation coefficient is estimated using the interGeneCorrelation() function in the limma R Package, as previously described (Wu and Smyth 2012). Considering that peptides from the same protein are generally positively correlated (see Subsection 3.4), we impose a non-negative constraint on correlation coefficients by setting negative correlations to zero.

The mean and standard deviation of peptide-wise *t*-statistics not in the POI are estimated using either the sample mean and SD or the sample median and MAD, respectively. In our algorithm, MAD is inherently scaled by 1.4826 to align with the SD. Mathematical details of the two-sided two-sample *t*-tests can be found in the Supplementary Information. The resulting *P*-values are adjusted using the Benjamini–Hochberg (BH) method (Benjamini and Hochberg 1995).

### 2.2 Estimation of inter-peptide correlation

Assume a set of *m* peptides belonging to a POI has equal pairwise correlation coefficients ρ. We consider the following linear mixed model:

$$y_{ij}=X_{ij}\beta_{i}+r_{j}+\varepsilon_{ij},$$

where $y_{ij}$ represents the log_2_-transformed abundance of the i-th peptide and j-th sample, $X_{ij}$ is the covariate, $r_{j}$ is the sample-associated random effect, and $\varepsilon_{ij}$ is the error term. We assume that $r_{j}$ and $\varepsilon_{ij}$ are identically, independently, and normally distributed, i.e. $r_{j}∼N\left(0,\;{\sigma_{r}}^{2}\right)$ and $\varepsilon_{ij}∼N\left(0,\;{\sigma_{e}}^{2}\right)$. The model is fitted to the data using the lmer() function in the lme4 R package (Bates *et al.* 2015).

Let $\sigma^{2}$ denote the total variance, i.e. $\sigma^{2}={\sigma_{r}}^{2}+{\sigma_{e}}^{2}$. Since all pairwise peptide correlation coefficients of the same protein are assumed to be equal, we know that the following equality holds:

$$\left[\begin{array}{cccc} \sigma^{2} & {\rho \sigma }^{2} & \cdots & {\rho \sigma }^{2}\\ {\rho \sigma }^{2} & \sigma^{2} & \cdots & {\rho \sigma }^{2}\\ \vdots & \vdots & \ddots & \vdots \\ {\rho \sigma }^{2} & {\rho \sigma }^{2} & \cdots & \sigma^{2} \end{array}\right]=\left[\begin{array}{cccc} {\sigma_{r}}^{2} & {\sigma_{r}}^{2} & \cdots & {\sigma_{r}}^{2}\\ {\sigma_{r}}^{2} & {\sigma_{r}}^{2} & \cdots & {\sigma_{r}}^{2}\\ \vdots & \vdots & \ddots & \vdots \\ {\sigma_{r}}^{2} & {\sigma_{r}}^{2} & \cdots & {\sigma_{r}}^{2} \end{array}\right]+\left[\begin{array}{cccc} {\sigma_{e}}^{2} & 0 & \cdots & 0\\ 0 & {\sigma_{e}}^{2} & \cdots & 0\\ \vdots & \vdots & \ddots & \vdots \\ 0 & 0 & \cdots & {\sigma_{e}}^{2} \end{array}\right].$$

Therefore, $\hat{\rho }$ can be estimated as:

$$\hat{\rho }=\frac{{\sigma_{r}}^{2}}{{\sigma_{r}}^{2}+{\sigma_{e}}^{2}},$$

from which we know that $\hat{\rho }$ must be non-negative. Similar to Wu *et al.*, we assume that the same correlations hold between peptide-wise *t*-statistics as between expression values, since correlations between peptide-wise *t*-statistics should be similar to those between the log-expression values in practice (Barry *et al.* 2008, Wu and Smyth 2012).

### 2.3 Simulations

The simulation was set up as follows. A total of 9000 peptides were generated with two groups of samples and $\frac{N}{2}$ replicates per group (N stands for the total number of samples). Without loss of generality, peptide-wise mean log_2_-transformed expression values, $\mu_{i}$ (i= 1, 2, …, 9000), were sampled from a standard normal distribution. Peptide-wise variances, ${\sigma_{i}}^{2}$, were all set to 1. Log_2_-transformed expression values were generated from a multivariate normal distribution with means equal to $\mu_{i}$ and a covariance matrix set as follows:

$$\left[\begin{array}{ccccccc} {\sigma_{1}}^{2} & \cdots & \rho \sigma_{1}\sigma_{m_{1}} & 0 & 0 & \cdots & 0\\ \vdots & \ddots & \vdots & \vdots & \vdots & \cdots & \vdots \\ \rho \sigma_{m_{1}}\sigma_{1} & \cdots & {\sigma_{m_{1}}}^{2} & 0 & 0 & \cdots & 0\\ 0 & \cdots & 0 & \ddots & 0 & \cdots & 0\\ 0 & \cdots & 0 & 0 & {\sigma_{\sum_{j=1}^{J-1}m_{j}+1}}^{2} & \cdots & \rho \sigma_{\sum_{j=1}^{J-1}m_{j}+1}\sigma_{\sum_{j=1}^{J}m_{j}}\\ \vdots & \cdots & \vdots & \vdots & \vdots & \ddots & \vdots \\ 0 & \cdots & 0 & 0 & \rho \sigma_{\sum_{j=1}^{J}m_{j}}\sigma_{\sum_{j=1}^{J-1}m_{j}+1} & \cdots & {\sigma_{\sum_{j=1}^{J}m_{j}}}^{2} \end{array}\right].$$

Here, J was the total number of proteins, $m_{j}$ was the number of peptides within the j-th protein (j= 1, 2, …, J), and ρ represented the inter-peptide correlation coefficients between an arbitrary pair of peptides within a protein. For simplicity, all simulated proteins were assumed to have the same ρ, although this assumption was not necessary for the peptide set test. The number of peptides belonging to the POI was allowed to vary and set to 3, 10, and 30, which corresponded to 3000, 900, and 300 proteins in total, respectively. Additional simulations were carried out assuming a mixture of proteins (1400 proteins with 3 peptides each, 360 proteins with 10 peptides each, and 40 proteins with 30 peptides each) to mimic a read-world proteomics dataset. ρ was set to either 0 or 0.05. The simulation was repeated 1000 times. Note that we assumed uniform inter-peptide correlation (ρ) across proteins in our simulations for simplicity. This is not a prerequisite for the competitive peptide set test, however, as our method only assumes identical inter-peptide correlations within a protein, not across proteins.

The *P*-value threshold was set to 0.05. To calculate the Type I error rate, the mean group difference was set to 0 for all proteins, and the proportion of significant proteins was counted towards the Type I error rate. To calculate power, 5% of proteins were allowed to be differentially expressed. The mean group difference was set to 0.5 for 2.5% of proteins and −0.5 for another 2.5% of proteins, and the proportion of significant proteins was counted towards the power. Note that the performance of MAD-based approaches was evaluated only in simulations where 5% of proteins were assumed to be differentially expressed. This choice was made because, in cases where no peptides are assumed to be active, the resulting *t*-statistics of peptides not in the POI should follow a Student’s *t*-distribution with very high degrees of freedom, approximating a normal distribution. Consequently, the MAD and SD estimators will produce similar results in this scenario.

### 2.4 Other approaches

To validate the competitive peptide set test, we applied several additional data analysis workflows for the sake of comparison. In aggregation-based workflows, peptide abundance values were collapsed into protein abundance using either summed peptide intensity or robust regression with M-estimation prior to performing a protein-level moderated *t*-test using limma (Supplementary Fig. S1) (Ritchie *et al.* 2015, Sticker *et al.* 2020). Summation of peptide intensity is one of the most widely used aggregation methods, while robust regression has been shown to outperform commonly used methods in several instances (Tyanova *et al.* 2016, Sticker *et al.* 2020, Gatto 2021) (https://lgatto.github.io/2021-03-15-RProt-online/sec-quant.html). If the proteome contains a mixture of proteins with variable numbers of peptides, we incorporate the log-transformed number of peptides as a covariate, adjusting the prior variances with the trend argument in the eBayes() function (Ritchie *et al.* 2015). In a self-contained peptide set test, a group of peptides from a protein was considered as a unit, independent of other proteins, and a single *P*-value was computed for the entire set based on the detection of significant differences in any peptide within the set (Wu *et al.* 2010). This test was conducted using the fry() function in limma (Wu *et al.* 2010).

### 2.5 Analysis of real-world experimental data

Contaminants, reverse hits, missing values, as well as systematic bias introduced during sample preparation and data generation can all pose threats to the integrity of subsequent data analysis. Therefore, real biological data used to validate our method were filtered and normalized prior to statistical analysis (see Supplementary Fig. S2 for details). We applied a 70% rule to filter missing values, retaining only analytes identified in at least 70% of samples. Our decision aligns with the original publications and common practices in proteomics, where researchers often opt for filtering strategies without imputation to handle missing values (Yang *et al.* 2015, De Marchi *et al.* 2016, Messner *et al.* 2020, Hristova *et al.* 2023, Lileikyte *et al.* 2023). Gene ontology enrichment analysis was performed using the enrichGO function() in the clusterProfiler R package (Yu *et al.* 2012, Wu *et al.* 2021).

## 3 Results

### 3.1 Peptide set test controls the type I error rate correctly

**Figure 2.**
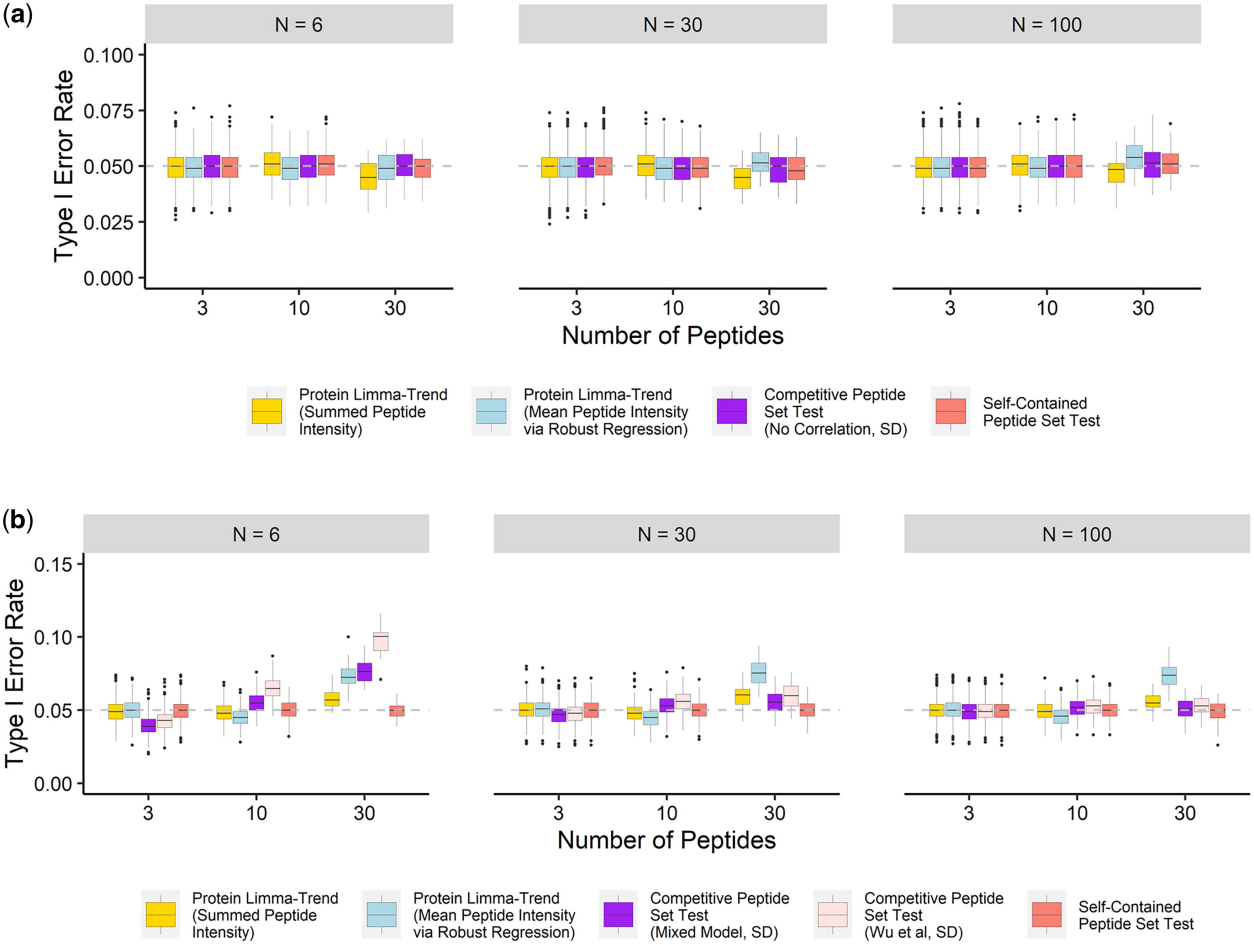
Type I error rates of different methods for differential protein expression analysis based on simulated data. Supplementary Table S2 provides detailed information about each method. The data were simulated with inter-peptide correlation coefficients (*ρ*) assumed to be (a) 0 or (b) 0.05. The simulated proteome consisted of a mixture of proteins, including 1400 3-peptide proteins, 360 10-peptide proteins, and 40 30-peptide proteins. No proteins were assumed to be differentially expressed. The number of samples (*N*) was set equal to 6, 30, or 100. The simulation was repeated 1000 times.

We evaluated the Type I error rate of the competitive peptide set test using simulated peptide abundance data. In our simulations, the number of samples, the number of peptides per protein, inter-peptide correlation coefficients, as well as the percentage of differentially expressed proteins, were allowed to vary. The resulting data were processed using the competitive peptide set test, conventional aggregation-based workflows, and self-contained peptide set test. In competitive peptide set tests, peptide-level *t*-statistics calculated using limma were compared between peptides that did and did not belong to the POI (Supplementary Fig. S1). Different methods to estimate inter-peptide correlation coefficients and the variance of peptides not in the POI were examined. A full list of simulated datasets, along with the methods applied to each dataset, can be found in Supplementary Table S1. Details of each method are provided in Supplementary Table S2. In the absence of inter-peptide correlation, all workflows demonstrated close control of the Type I error rate around 0.05 when assuming that all proteins contained the same number of peptides (Supplementary Fig. S3a). Comparable specificity levels were upheld in more realistic settings where the proteome contained a mixture of proteins (Fig. 2a).

In the presence of inter-peptide correlation, the competitive peptide set tests correctly controlled the Type I error rate, except for the 30 peptide-set, *N* = 6 case (Fig. 2b and Supplementary Fig. S3b). This discrepancy may arise from the need for a relatively large sample size to provide a close estimation of the true correlation coefficient. Regarding correlation estimation, the approach by Wu *et al.* is faster and makes fewer distribution assumptions but yields higher Type I error rates than the mixed model approach for small sample sizes (Fig. 2b and Supplementary Fig. S3b) (Wu and Smyth 2012). This is likely due to interGeneCorrelation() risking an even greater underestimation of the average inter-peptide correlation compared to the mixed model approach (Supplementary Fig. 4). Additionally, interGeneCorrelation() does not allow NAs, potentially complicating the analysis of real-world proteomics data.

It is noteworthy that, unlike peptide set tests, the limma-trend workflow utilizing robust regression to aggregate peptides failed to control Type I error rates correctly for 30 peptide-sets in mixture proteomes, regardless of sample size (Fig. 2b). This may be because limma-trend tends to underestimate the variance of peptide-rich proteins (i.e. proteins matched to many peptides) due to the asymmetrical distribution of protein variance potentially caused by robust regression-based aggregation (Supplementary Fig. S5).

### 3.2 Peptide set test demonstrates improved power

To evaluate the power of the peptide set tests, we conducted additional simulations, allowing 5% of proteins in the proteome to be differentially expressed. As expected, the power of the tests increased with the number of peptides per protein, indicating that peptide set tests are more sensitive for peptide-rich proteins than for peptide-sparse ones (Supplementary Figs S6 and S7). The self-contained test exhibited good power, particularly for relatively large sample sizes, but for *N* = 6, its performance was suboptimal, regardless of inter-peptide correlations (Supplementary Figs S6 and S7). Compared to the self-contained test, a competitive peptide set test demonstrated superior power to detect protein changes, especially for small sample sizes (Supplementary Figs S6 and S7), without compromising the Type I error rate in most cases (Supplementary Figs S8 and S9). The MAD-based approach used to estimate the variance of peptides not in the POI controlled the Type I error rate more closely around 0.05 than the SD-based approach (Supplementary Figs S8 and S9). Although most proteins in the proteome were inactive, the minority of active proteins could still stand out as outliers, necessitating robust estimators such as sample MAD to accurately estimate the variability of peptides that did not belong to the POI.

### 3.3 Peptide set test demonstrates balanced control of false discovery rates and true positive rates

To evaluate the control of false discovery rates (FDRs) and true positive rates (TPRs), we further compared the performance of each method after adjusting *P*-values for multiple testing using the BH method (Benjamini and Hochberg 1995). Additionally, *P*-values from the peptide set test were exploratively adjusted using the more conservative Benjamini-Yekutieli (BY) method to account for potential dependency in multiple hypothesis testing (Benjamini and Yekutieli 2001). Details of FDR and TPR calculation are provided in the Supplementary Methods.

**Figure 3.**
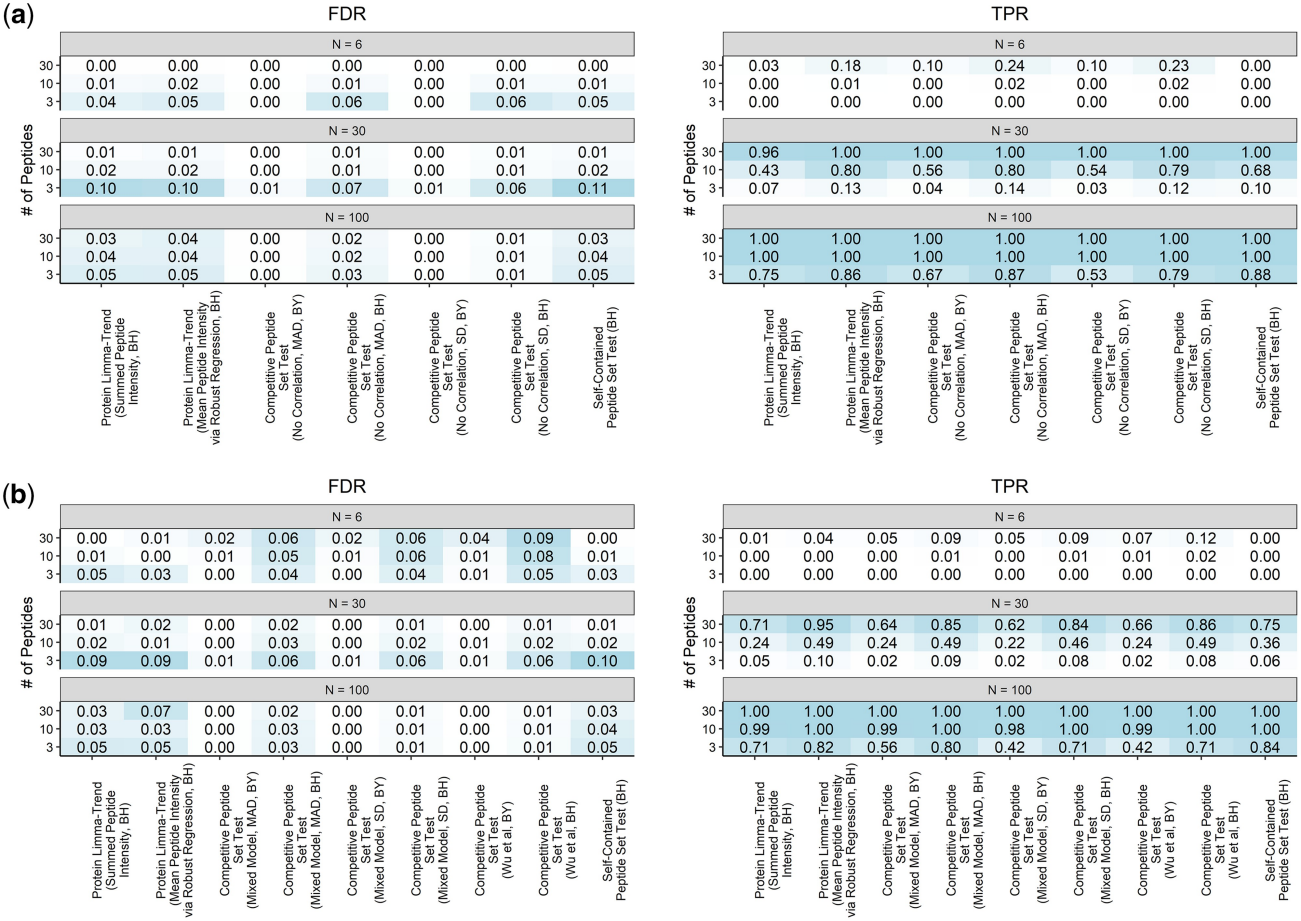
False discovery rates (FDRs) and true positive rates (TPRs) of different methods for differential protein expression analysis based on simulated data. Supplementary Table S2 provides detailed information about each method. The data were simulated with inter-peptide correlation coefficients (*ρ*) assumed to be (**a**) 0 or (**b**) 0.05. The simulated proteome contained a mixture of proteins, including 1400 3-peptide proteins, 360 10-peptide proteins, and 40 30-peptide proteins. Five percent of the proteins were assumed to be differentially expressed. The number of samples (*N*) was set equal to 6, 30, or 100. The simulation was repeated 1000 times.

With an adjusted *P*-value threshold of 0.05, the BY method appeared overly conservative, leading to reduced power compared to the BH method (Fig. 3). The MAD-based competitive peptide set test coupled with BH correction yielded one of the highest TPRs, especially for 30-peptide proteins. At the same time, it efficiently controlled FDRs in most cases, regardless of inter-peptide correlations (Fig. 3).

Overall, the mixed-model approach used to estimate inter-peptide correlations, combined with the sample MAD-based estimator and BH correction, demonstrated the best combination of TPRs and FDRs among all versions of competitive peptide set tests examined. Henceforth, this method is referred to as the “peptide set test.”

### 3.4 Proteomics datasets show positive inter-peptide correlations

Peptides from the same protein are generally anticipated to exhibit positive correlation. To assess the strength of inter-peptide correlations in well-controlled experiments, we analyzed a previously published proteomics study involving breast cancer patients with distinct responses to tamoxifen treatment (De Marchi *et al.* 2016). The study involved data collected from two medical centers: the EMC dataset comprised 56 tissue samples from Erasmus MC University Medical Center, Rotterdam, Netherlands, and the NKI-AVL + RUMC dataset consisted of 41 samples from the National Cancer Institute—Antoni van Leeuwenhoek hospital, Amsterdam and 15 from Radboud University Medical Center, Nijmegen (De Marchi *et al.* 2016). All patients underwent surgery of their primary tumor and received tamoxifen as first-line therapy after developing recurrent disease (De Marchi *et al.* 2016). Treatment outcome was defined based on time to progression, with disease progression ≤6 months and >6 months after start of tamoxifen administration defined as poor and good outcomes, respectively (De Marchi *et al.* 2016). Details of the study, including sample collection, sample preparation, and LC-MS/MS analysis can be found in De Marchi *et al.* (2016).

To eliminate outcome-dependent effects, averaged inter-peptide correlations were computed for all proteins within each treatment outcome group in each dataset. The mean averaged inter-peptide correlation reached 0.57, 0.59, 0.61, and 0.54 within hormone-sensitive and hormone-resistant patient cohorts in the EMC and NKI-AVL + RUMC datasets, respectively (Supplementary Fig. S10). This suggests that positive inter-peptide correlations are typical for peptides belonging to the same protein.

### 3.5 Peptide set test outperforms aggregation-based methods in a spike-in experiment

**Figure 4.**
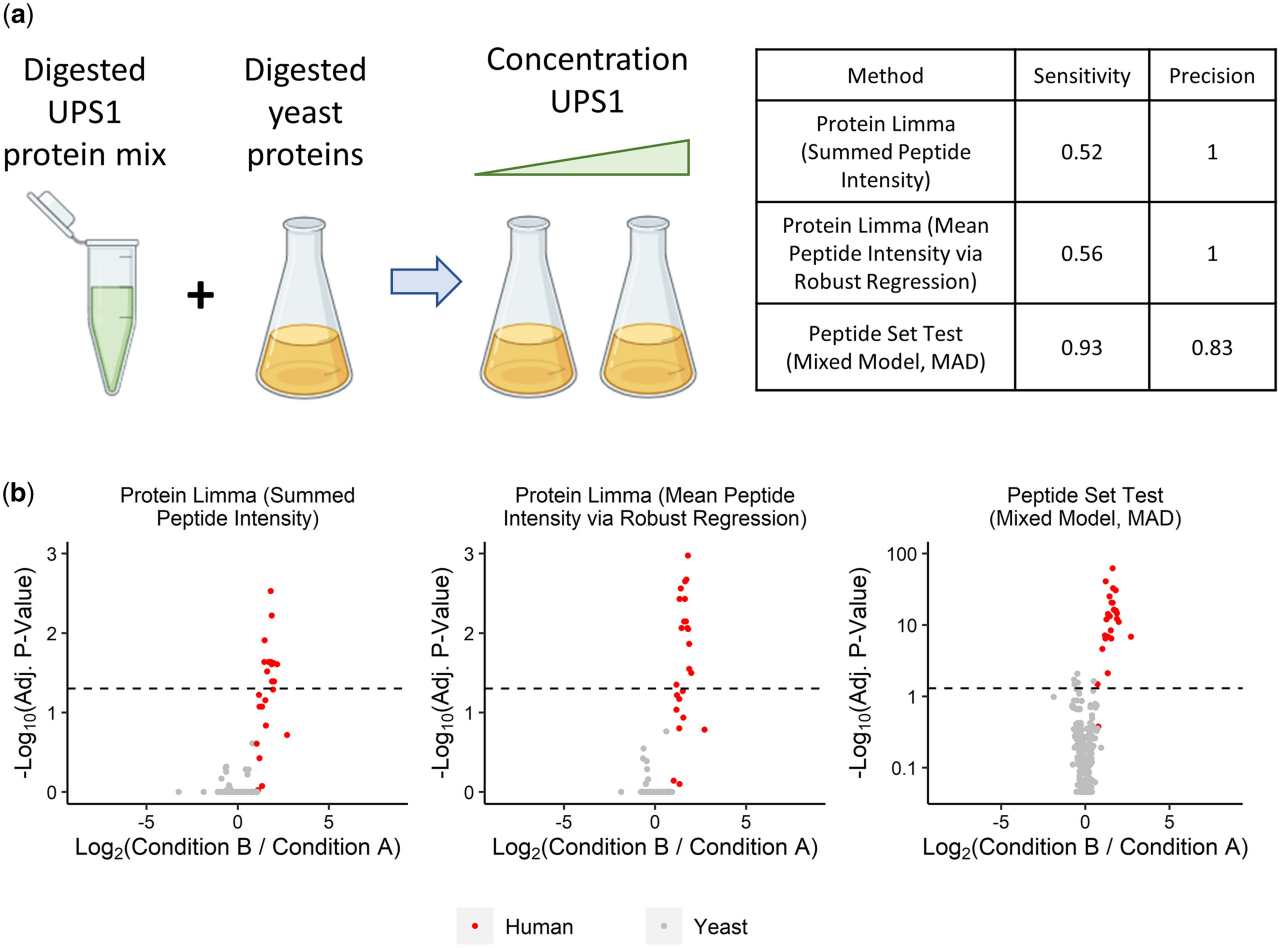
Comparison of the peptide set test to traditional aggregation-based methods based on a CPTAC spike-in experiment. (a) Description of the CPTAC spike-in experiment, along with the sensitivity of three statistical workflows using an adjusted *P*-value threshold of 0.05. In this study, human universal protein standards (UPS1) were spiked into yeast at two different concentrations. The peptide set test was compared against aggregation-based methods to evaluate its ability to detect differentially expressed proteins. Supplementary Table S2 provides detailed information about each method. (b) Volcano plots highlighting the differentially expressed proteins detected by each statistical workflow. Proteins with an adjusted *P*-value larger than 0.9 in the peptide set test were converted to 0.9 for visualization purposes. Dashed horizontal lines indicate the adjusted *P*-value threshold of 0.05.

To further validate our method, we compared the performance of peptide set tests with traditional aggregation-based approaches using a subset of data from the CPTAC spike-in study 6, which involved the Sigma UPS1 standard spiked into a constant yeast protein background at 6.7 fmol/µl and 20 fmol/μl, respectively (Fig. 4a) (Paulovich *et al.* 2010). The experiments for each condition were carried out in three replicates (*N* = 6). In the resulting six samples, all human proteins were considered differentially expressed (active), while all yeast proteins were considered inactive. The corresponding MaxQuant-processed data were provided as a part of the msdata R package (Tyanova *et al.* 2016, Neumann *et al.* 2019).

We re-analyzed the peptide-level data using the same three workflows described earlier: peptide set test, summation-based limma workflow, and robust regression-based limma workflow (Ritchie *et al.* 2015, Sticker *et al.* 2020, Gatto 2021). *P*-values were adjusted using the BH method (Benjamini and Hochberg 1995). Although simulation suggested that the peptide set test might inflate the Type I error rate in the 30 peptide-set, *N* = 6 case (Fig. 2b and Supplementary Fig. S3b), the majority (91%) of proteins in this dataset contained 10 or fewer peptides. Therefore, it is unlikely that the performance of the peptide set test would be strongly affected by the small sample size.

In this dataset, 1150 proteins contained at least one peptide that was present in more than 67% of all samples. These proteins were considered to be reliably quantified and used for subsequent statistical analysis. Since we had prior knowledge of which proteins were changing (only the human ones), we could calculate and compare the sensitivity and precision of each workflow. With an adjusted *P*-value threshold of 0.05, the peptide set test identified 25 out of 27 reliably quantified human proteins as differentially expressed, achieving the highest sensitivity among all methods tested (Fig. 4). Five yeast proteins were incorrectly identified as active by the peptide set test, resulting in a precision of 83% (Fig. 4). Despite a slight decrease in precision, neither aggregation-based method was able to achieve a level of sensitivity comparable to that of the peptide set test.

### 3.6 Peptide set test reveals widespread proteome changes in tamoxifen-resistant breast cancer

**Figure 5.**
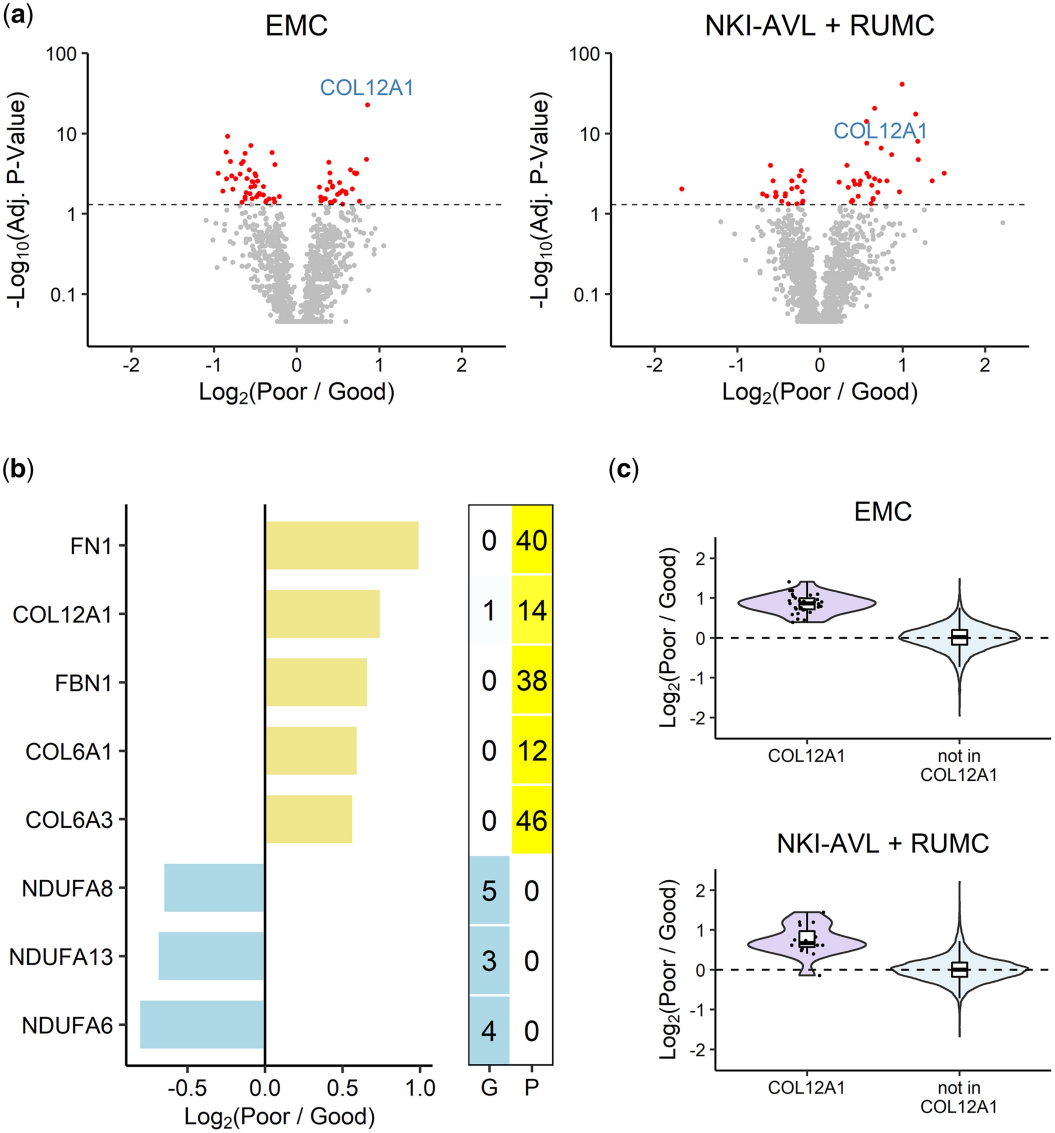
Tamoxifen resistance-associated protein signatures in breast cancer patients. (a) Volcano plots highlighting protein expression changes in tamoxifen-resistant breast cancer patients based on the results of the peptide set test. Proteins with an adjusted *P*-value larger than 0.9 were converted to 0.9 for visualization purposes. Dashed horizontal lines indicate an adjusted *P*-value threshold of 0.05. Differentially regulated proteins are highlighted in red. COL12A1 is labeled. (b) Bar plots showing the average log_2_ peptide fold changes of proteins involved in extracellular matrix organization and oxidative phosphorylation in patients who manifested good (G) and poor (P) outcomes to tamoxifen treatment. The heat maps illustrate the number and color-coded proportion of peptides that are upregulated (blue) and downregulated (yellow). (c) Violin plots highlighting the log_2_ fold changes of COL12A1 peptides in the EMC and NKI-AVL + RUMC cohorts. Black dots represent the log_2_ fold change of each peptide. Boxes highlight the quartiles.

To demonstrate the ability of the peptide set test to recover biological insights, we applied our method to analyze the breast cancer dataset described in Subsection 3.4. Protein signatures represent lists of proteins whose differential expression is associated with a specific phenotype. To identify protein signatures specific to the tamoxifen-resistant breast cancer cohort, we compared the tumor proteomes of patients with good and poor outcomes to tamoxifen. Analysis of the data using the peptide set test revealed 73 and 56 differentially regulated proteins (ie, proteins with an adjusted *P*-value less than 0.05) in the EMC and NKI-AVL + RUMC cohorts, respectively (Fig. 5a). For comparison, we also fitted separate linear models to the EMC and NKI-AVL + RUMC data using limma after peptide aggregation (Dahan *et al.* 2015, Mutai *et al.* 2022). In contrast to the peptide set test, the robust regression approach identified only 1 and 4 significantly regulated proteins in the EMC and NKI-AVL + RUMC cohorts, respectively (Supplementary Fig. S11). The summation approach did not identify any significant proteins in either cohort (Supplementary Fig. S11).

Gene ontology biological process enrichment analysis of the two protein lists derived from the peptide set test revealed that the EMC protein signature was enriched in oxidative phosphorylation, as components of the NADH dehydrogenase (ubiquinone) 1 alpha subcomplex, namely NDUFA8, NDUFA13, and NDUFA6, were downregulated in hormone-resistant patients. The NKI-AVL + RUMC signature was enriched in extracellular matrix (ECM) organization, as ECM proteins, including FN1, COL12A1, FBN1, COL6A1, and COL6A3 were found to be upregulated (Fig. 5b). In particular, COL12A1, a critical regulator of tumor matrix organization, was more abundant in tamoxifen-resistant patients in both the EMC and NKI-AVL + RUMC cohorts, with average log_2_ fold changes of 0.86 (FDR = 1.36 × 10^−22^) and 0.74 (FDR = 2.07 × 10^−7^), respectively (Fig. 5c). The consistent upregulation of COL12A1 in both cohorts confirmed its association with tamoxifen resistance. Interestingly, neither the summation-based nor robust regression-based limma workflow ranked COL12A1 highly based on its *P*-value, especially in the EMC dataset (Supplementary Fig. S11).

To assess the applicability of the peptide set test at the precursor level, we reprocessed the raw proteomics data in MaxQuant using the provided parameter settings (De Marchi *et al.* 2016). The proteome-wide log_2_ fold changes calculated based on the precursor-level data and the peptide-level data exhibit a 97% correlation in both the EMC and NKI-AVL + RUMC datasets (Supplementary Fig. S12). This indicates the effectiveness of our method at both precursor and peptide levels.

## 4 Discussion

In this work, we have developed a competitive peptide set test that identifies differentially expressed proteins by employing a statistical framework that considers both the magnitude of fold change and the number of peptide identifications. It reformulates the question of identifying differentially expressed proteins into a comparison between one protein and the rest of the peptidome. Since the number of protein identifications is typically much larger than the number of samples in a proteomics experiment, the degrees of freedom are increased by this reformulation. Our method can be valuable for discovering novel biomarkers and prioritizing drug targets, especially when the direct application of statistical analysis to protein data fails to provide substantial insights.

Our approach offers a large flexibility in formulating the peptide-level statistical model, allowing for compatibility with a wide range of meaningful test statistics (e.g., *t*-statistics, log_2_ fold change) derived from the model. We have demonstrated that the competitive peptide set test achieves superior power compared to the self-contained peptide set test and traditional aggregation-based approaches while maintaining the correct Type I error rate in nearly all cases. Aggregation-based methods, which aggregate correlated yet seemingly independent peptides, tend to overestimate protein variance. While limma-trend can correct variance estimation by considering peptide numbers, real-world scenarios pose challenges in enumerating all relevant covariates, such as varying inter-peptide correlations across proteins. In contrast, the competitive peptide set test addresses this issue by directly accounting for inter-peptide correlations.

To control Type I error rates effectively, we have adopted MAD as a robust variance estimator. Although estimating associated degrees of freedom with MAD might be challenging, the large degrees of freedom for peptides not in the POI minimally impact downstream hypothesis testing, making an accurate estimation unnecessary. Wilcoxon rank-sum tests, which relax the distribution assumption, present as a viable alternative to the two-sided *t*-test. However, incorporating inter-peptide correlations into Wilcoxon rank-sum tests requires a normal approximation, making them less reliable for proteins with relatively few peptide identifications (Wu and Smyth 2012).

In handling missing values in real-world proteomics data, we have chosen filtering over imputation. Imputation methods often rely on assumptions about the distribution of missing values, and these assumptions might not hold true for proteomics data. Conversely, treating missing values as NA avoids introducing potentially biased or inaccurate imputed values. Leaving missing values as NA has minimal impact on the estimation of correlations or the number of peptides that can be included in the analysis. This is because both the mixed model-based approach for estimating inter-peptide correlations and the competitive peptide set test are designed to function in the presence of NA, under the assumption that values are missing at random. It is important to note that we did not address cases where values are missing not at random. Future research endeavors could explore these scenarios.

Using the peptide set test, we have identified a protein signature associated with tamoxifen resistance that may shed light on the pathophysiological processes contributing to a higher risk of resistance to endocrine therapy in breast cancer. Tumor samples from hormone-resistant patients in the EMC cohort exhibited impaired oxidative phosphorylation, which could trigger a metabolic shift in cancer cells towards enhanced aerobic glycolysis or glutaminolysis, as observed in various types of cancer, including tamoxifen-resistant breast cancer (Woo *et al.* 2015, Steifensand *et al.* 2021, Wang *et al.* 2022). Conversely, dysregulation of ECM organization in the NKI-AVL + RUMC hormone-resistant cohort suggested an invasive cancer phenotype (Bonnans *et al.* 2014). The abundance of COL12A1 peptides were higher in tamoxifen-resistant patients in both cohorts. Previous studies have shown that collagen XII secreted by cancer-associated fibroblasts can alter collagen I organization in the tumor microenvironment, increasing collagen I fiber density, bundle width, and fiber linearity (Papanicolaou *et al.* 2022). Another study revealed that an increasingly aligned orientation of collagen I fibrils could promote tumor cell proliferation in an ER-independent manner (Reyes-Ramos *et al.* 2021). Therefore, it is reasonable to hypothesize that by regulating matrix assembly, collagen XII may not only create a pro-invasive environment that facilitates metastatic dissemination, as previously described (Papanicolaou *et al.* 2022), but also promote ER independence, leading to tamoxifen resistance. Importantly, COL12A1 was ranked much lower based on the *P*-values obtained from aggregation-based approaches (Supplementary Fig. S11). This protein would unlikely have been prioritized for follow-up investigation if we had relied on traditional approaches to analyze the data.

Our results suggest several opportunities for further improving the performance of the peptide set test through refined statistical modeling. One assumption is that all proteins have approximately the same variance in peptide-wise *t*-statistics. It also assumes that the majority of the proteome is not differentially expressed. These two assumptions collectively ensure that the variances of *t*-statistics for peptides belonging to a protein and those that do not are approximately equal, which is necessary for justifying the application of the two-sample *t*-test. While two-sample *t*-tests can tolerate moderate departures from homogeneity of variance (Dean and Voss 1999), a minority of proteins might exhibit excessively large or small variances. Estimating individual variances using an empirical Bayes approach followed by a Welch’s *t*-test may offer a better alternative for such proteins.

Another area for improvement is the estimation of inter-peptide correlation. As shown in Fig. 2b, the peptide set test resulted in an inflated Type I error rate in the 30 peptide-set, *N* = 6 case. This suggests that our method is likely to perform better in studies with medium-to-large-sized cohorts or small-sized cohorts with relatively short liquid chromatography (LC) gradients (shorter LC gradients typically lead to fewer peptide identifications per protein). More sophisticated estimators of inter-peptide correlation are needed to overcome the limitations of sample size. Moreover, our mixed model approach assumes that peptides originating from the same protein have equal inter-peptide correlation coefficients. This assumption may not hold if a protein has multiple isoforms, as subgroups of peptides within this protein may represent different biology. Under such a circumstance, one may consider clustering peptides based on correlation.

In addition to the above assumptions, the peptide set test implemented here also assumes that the average log_2_ fold change of the peptidome is approximately zero, meaning that the fractions of up- and downregulated proteins are comparable. Although this assumption is common in discovery proteomics, the complexity of protein mixtures obtained from pull-down assays or analysis of secretomes typically exhibits stronger variation between control and treatment samples. Without this assumption, we might fail to reject the null hypothesis even if most of the peptides in the peptidome are differentially expressed. It is worth noting that the competitive peptide set test is intended to complement, rather than replace, the traditional aggregation-based methods and the self-contained peptide set test, as the hypotheses being tested in these methods are fundamentally different. A traditional self-contained *t*-test determines whether the mean protein abundance is equal between two groups. A self-contained peptide set test examines whether any peptide belonging to a protein exhibits significant differences without reference to other proteins, whereas a competitive peptide set test compares the fold change of a protein represented by its peptides against the rest of the peptidome.

Finally, it is worth noting that while our method demonstrates increased statistical power, it only evaluates the statistical significance, not the practical significance of changes in protein expression. In reality, a protein is likely to spark interest in a scientific context only if its expression level changes substantially. To mitigate this issue, one may consider imposing a cutoff on the log_2_ fold changes of proteins or comparing the peptide-wise log_2_ fold changes instead of the *t*-statistics. Novel methods are needed to test for differential protein expression that is both statistically significant and biologically meaningful.

In conclusion, we have developed a sensitive peptide set test that adopts a peptide-centric strategy for prioritizing biologically important protein candidates. This test enables us to gain insights into the biological mechanisms associated with tamoxifen resistance in breast cancer patients. Our work demonstrates the great potential of this approach in accelerating biological discovery and facilitating clinical translation.

## Supplementary Material

btae270_Supplementary_Data

## Data Availability

The MaxQuant search output generated from the spike-in experiment has been previously published and is provided as a part of the msdata R package (https://bioconductor.org/packages/release/data/experiment/html/msdata.html) (Paulovich *et al.* 2010, Neumann *et al.* 2019). The MaxQuant search output generated from the breast cancer study has also been previously published and can be accessed at the ProteomeXchange Consortium via the PRIDE partner repository with dataset identifiers PXD000484 and PXD000485 (https://www.proteomexchange.org/) (Vizcaino *et al.* 2013, De Marchi *et al.* 2016).

## Code availability

The peptide set tests and aggregation-based workflows, along with the scripts used for generating simulated data and analysing data from the spike-in experiment and breast cancer study, are available as a part of the PepSetTest R package. The PepSetTest R package, including all the necessary code, can be found at https://github.com/JmWangBio/PepSetTest.
