# Supplementary material for: Peptide set test: a peptide-centric strategy to infer differentially expressed proteins
Source: Bioinformatics. 2024 Apr 17;40(5):btae270. doi: 10.1093/bioinformatics/btae270 (PMC11074007; doi:10.1093/bioinformatics/btae270)
Supplement: btae270_Supplementary_Data [file btae270_supplementary_data.pdf]

Peptide Set Test: a Peptide-Centric Strategy to Infer Differentially Expressed Proteins

Junmin Wang[1,†], **Steven Novick**[2]

[1] *Data Sciences and Quantitative Biology, Discovery Sciences, Biopharmaceuticals R&D, AstraZeneca, Gaithersburg, Maryland*

[2] *Global Statistical Sciences, Eli Lilly, Indianapolis, Indiana*

[†] *Corresponding author: Junmin Wang (junmin.wang@astrazeneca.com)*

Supplementary Information

- Supplementary Methods
- Supplementary Tables
- Supplementary Figures
- Supplementary References

Supplemental Methods

Steps of the two-sided two-sample t-test

The two-sided two-sample t-test operates in the following steps.

1. State null and alternative hypotheses:

$$H_0: \mu_{t_P} = \mu_{t_{P_C}}$$

$$H_a: \mu_{t_P} \neq \mu_{t_{P_C}}$$

2. Set the significance level: $\alpha = 0.05$.
3. Calculate t-statistics.

   If peptides are **NOT** correlated, t-statistics can be expressed as:

$$t = \frac{\bar{t}_P - \bar{t}_{P_C}}{s_p\sqrt{\frac{1}{m_P} + \frac{1}{m_{P_C}}}}$$

   where $\bar{t}_P$ is the mean t-statistic of peptides belonging to the protein of interest (POI), and $\bar{t}_{P_C}$ is the mean or the median t-statistic of peptides that do not belong to the POI. m_P and m_{P_C} are the number of peptides in each set. s_p is the pooled standard deviation, i.e.,

$$s_p = \sqrt{\frac{(m_P - 1)s_P^2 + \left(m_{P_C} - 1\right)s_{P_C}^2}{m_P + m_{P_C} - 2}}$$

   where s_P is the sample standard deviation of peptide-wise t-statistics belonging to the POI. s_{P_C} is either the sample standard deviation (SD) or the sample median absolute deviation (MAD) of peptide-wise t-statistics not in the POI scaled by 1.4826.

   If peptides from the same protein are **correlated**, t-statistics can be expressed as:

$$t = \frac{\bar{t}_P - \bar{t}_{P_C}}{s_p\sqrt{\frac{1 + (m_P - 1)\hat{\rho}_P}{m_P} + \frac{1}{m_{P_C}}}}$$

where $\bar{t}_P$, $\bar{t}_{P_C}$, m_P, m_{P_C}, and s_p are defined the same as previously described. If all peptides of protein P are assumed to have equal pairwise correlation coefficients, $\hat{\rho}_P$ is estimated by the linear mixed model described in the main text. Otherwise, $\hat{\rho}_P$ is an estimator of the average inter-peptide correlation coefficient $\bar{\rho}_P$, which calculation has been described in a separate publication[1].

4. Accept H_0 if $|t| < \left|t_{\frac{\alpha}{2},(m_1+m_2-2)}\right|$; reject H_0 if $|t| > \left|t_{\frac{\alpha}{2},(m_1+m_2-2)}\right|$.

Calculation of false discovery rates and true positive rates

In a simulated proteome consisting of J proteins, each protein j is associated with a number of peptides m_j, a real mean-group difference δ_j, and an adjusted p-value P_j. With an adjusted p-value threshold of 0.05, the false discovery proportion (FDP) of m^*-peptide proteins is defined as the proportion of false discoveries among total rejections, i.e.,

$$FDP = \frac{\sum_{j\in\{j|j=1,2,\dots,J\ \cap\ m_j=m^*\ \cap\ P_j<0.05\ \cap\ \delta_j=0\}} 1}{max\left\{1, \sum_{j\in\{j|j=1,2,\dots,J\ \cap\ m_j=m^*\ \cap\ P_j<0.05\}} 1\right\}}.$$

False discovery rate (FDR) is defined as the average FDP across 1000 simulations.

The true positive proportion (TPP) of m^*-peptide proteins is defined as the proportion of true discoveries among total positives, i.e.,

$$TPP = \frac{\sum_{j\in\{j|j=1,2,\dots,J\ \cap\ m_j=m^*\ \cap\ \delta_j\neq 0\ \cap\ P_j<0.05\}} 1}{max\left\{1, \sum_{j\in\{j|j=1,2,\dots,J\ \cap\ m_j=m^*\ \cap\ \delta_j\neq 0\}} 1\right\}}.$$

True positive rate (TPR) is defined as the average TPP across 1000 simulations.

Supplemental Tables

Figures	Simulation			Competitive Peptide Set Test	
	Proteome	Group Mean Difference	ρ	$\hat{\rho}$	s_{P_C}
Supplementary Fig. 3a	3000 x 3-peptide proteins, 900 x 10-peptide proteins, or 300 x 30-peptide proteins	0	0	NA	Sample SD
Supplementary Fig. 3b	3000 x 3-peptide proteins, 900 x 10-peptide proteins, or 300 x 30-peptide proteins	0	0.05	Mixed model or Wu et al	Sample SD
Supplementary Fig. 6a, Supplementary Fig. 8a	3000 x 3-peptide proteins, 900 x 10-peptide proteins, or 300 x 30-peptide proteins	0 (95%), 0.5 (2.5%), and -0.5 (2.5%)	0	NA	Sample SD or sample MAD (x1.4826)
Supplementary Fig. 6b, Supplementary Fig. 8b	3000 x 3-peptide proteins, 900 x 10-peptide proteins, or 300 x 30-peptide proteins	0 (95%), 0.5 (2.5%), and -0.5 (2.5%)	0.05	Mixed model or Wu et al	Sample SD or sample MAD (x1.4826)
Fig. 2a	1400 x 3-peptide proteins, 360 x 10-peptide proteins, and 40 x 30-peptide proteins	0	0	NA	Sample SD
Fig. 2b	1400 x 3-peptide proteins, 360 x 10-peptide proteins, and 40 x 30-peptide proteins	0	0.05	Mixed model or Wu et al	Sample SD
Supplementary Fig. 7a, Supplementary Fig. 9a, Fig. 3a	1400 x 3-peptide proteins, 360 x 10-peptide proteins, and 40 x 30-peptide proteins	0 (95%), 0.5 (2.5%), and -0.5 (2.5%)	0	NA	Sample SD or sample MAD (x1.4826)
Supplementary Fig. 7b, Supplementary Fig. 9b, Fig. 3b	1400 x 3-peptide proteins, 360 x 10-peptide proteins, and 40 x 30-peptide proteins	0 (95%), 0.5 (2.5%), and -0.5 (2.5%)	0.05	Mixed model or Wu et al	Sample SD or sample MAD (x1.4826)

Supplementary Table 1. Complete list of figures generated from the simulations along with the corresponding simulation parameters and estimators used in the competitive peptide set tests.

Method	Detail
Protein Limma and Protein Limma-Trend (Summed Peptide Intensity)	Peptide abundance values are collapsed into protein abundance via summed peptide intensity prior to performing protein-level moderated t-tests by limma[2]. Limma-trend adjusts prior variances by incorporating the log-transformed number of peptides as a covariate.
Protein Limma and Protein Limma-Trend (Mean Peptide Intensity via Robust Regression)	Peptide abundance values are collapsed into protein abundance via robust regression prior to performing protein-level moderated t-tests by limma[2]. Limma-trend adjusts prior variances by incorporating the log-transformed number of peptides as a covariate.
Competitive Peptide Set Test (No Correlation, SD)<br>Fig. 2a, Fig. 3a, Supplementary Fig. 3a, Supplementary Fig. 6a, Supplementary Fig. 7a, Supplementary Fig. 8a, Supplementary Fig. 9a	Inter-peptide correlation coefficients are neither estimated nor accounted for in the two-sample t-test. Sample standard deviation is adopted to estimate the standard deviation of peptide-wise t-statistics not in the protein of interest.
Competitive Peptide Set Test (Mixed Model, SD)<br>Fig. 2b, Fig. 3b, Supplementary Fig. 3b, Supplementary Fig. 4, Supplementary Fig. 6b, Supplementary Fig. 7b, Supplementary Fig. 8b, Supplementary Fig. 9b	Inter-peptide correlation coefficients are estimated via the mixed model approach and accounted for in the two-sample t-test. Sample standard deviation is adopted to estimate the standard deviation of peptide-wise t-statistics not in the protein of interest.
Competitive Peptide Set Test (Wu et al, SD)<br>Fig. 2b, Fig. 3b, Supplementary Fig. 3b, Supplementary Fig. 4, Supplementary Fig. 6b, Supplementary Fig. 7b, Supplementary Fig. 8b, Supplementary Fig. 9b	Inter-peptide correlation coefficients are estimated via the original approach proposed by Wu et al (2012), *Nucleic Acids Research*[1]. Sample standard deviation is adopted to estimate the standard deviation of peptide-wise t-statistics not in the protein of interest.
Competitive Peptide Set Test (No Correlation, MAD)<br>Fig. 3a, Supplementary Fig. 6a, Supplementary Fig. 7a, Supplementary Fig. 8a, Supplementary Fig. 9a	Inter-peptide correlation coefficients are neither estimated nor accounted for in the two-sample t-test. Sample median absolute deviation scaled by 1.4826 is adopted to estimate the standard deviation of peptide-wise t-statistics not in the protein of interest.
Competitive Peptide Set Test (Mixed Model, MAD)<br>Fig. 3b, Fig. 4, Fig. 5, Supplementary Fig. 6b, Supplementary Fig. 7b, Supplementary Fig. 8b, Supplementary Fig. 9b	Inter-peptide correlation coefficients are estimated via the mixed model approach and accounted for in the two-sample t-test. Sample median absolute deviation scaled by 1.4826 is adopted to estimate the standard deviation of peptide-wise t-statistics not in the protein of interest.
Self-Contained Peptide Set Test<br>Fig. 2, Fig. 3, Supplementary Fig. 3, Supplementary Figs. 6 - 9	A set of peptides belonging to a protein is treated as a unit without reference to other proteins. A single-p-value is evaluated for the entire set based on the detection of significant differences in any peptide within the set[3].

Supplementary Table 2. Detailed information about the methods shown in the legends of Figs. 2–5 and Supplementary Figs. 3–9.

Supplementary Figures

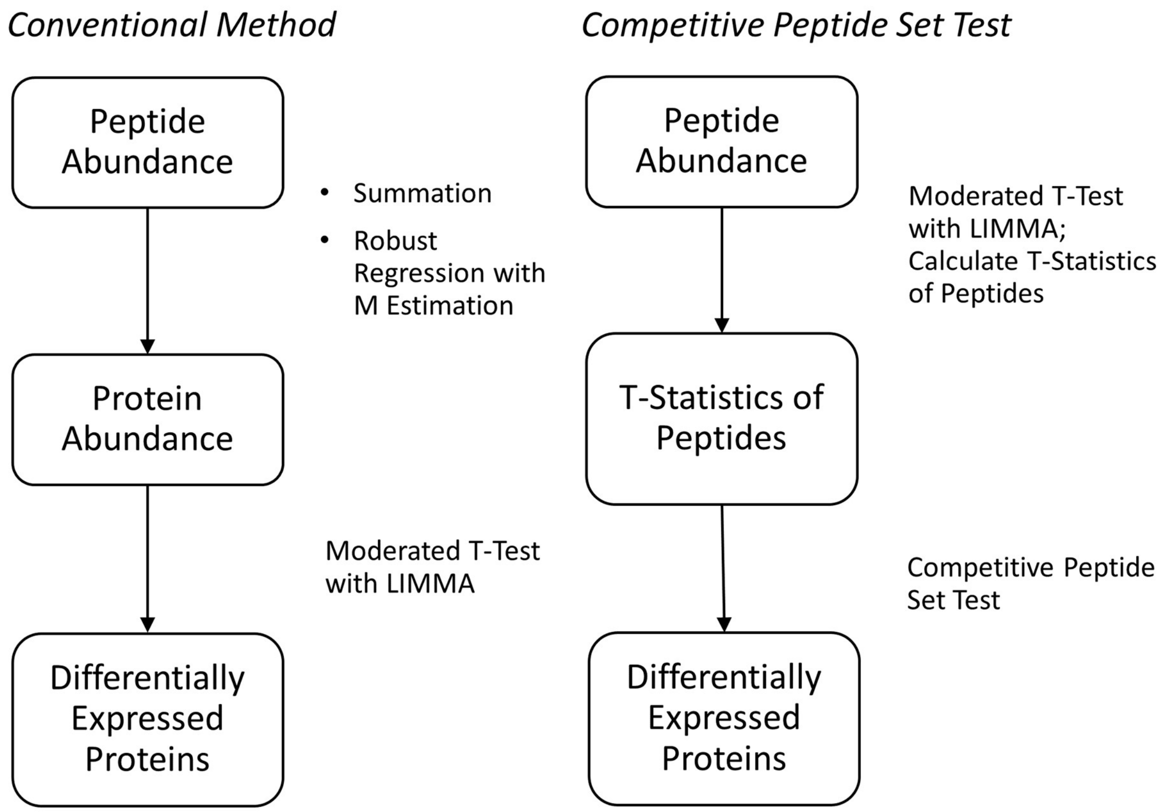

Supplementary Fig. 1. Diagram comparing conventional aggregation-based methods and the competitive peptide set test.

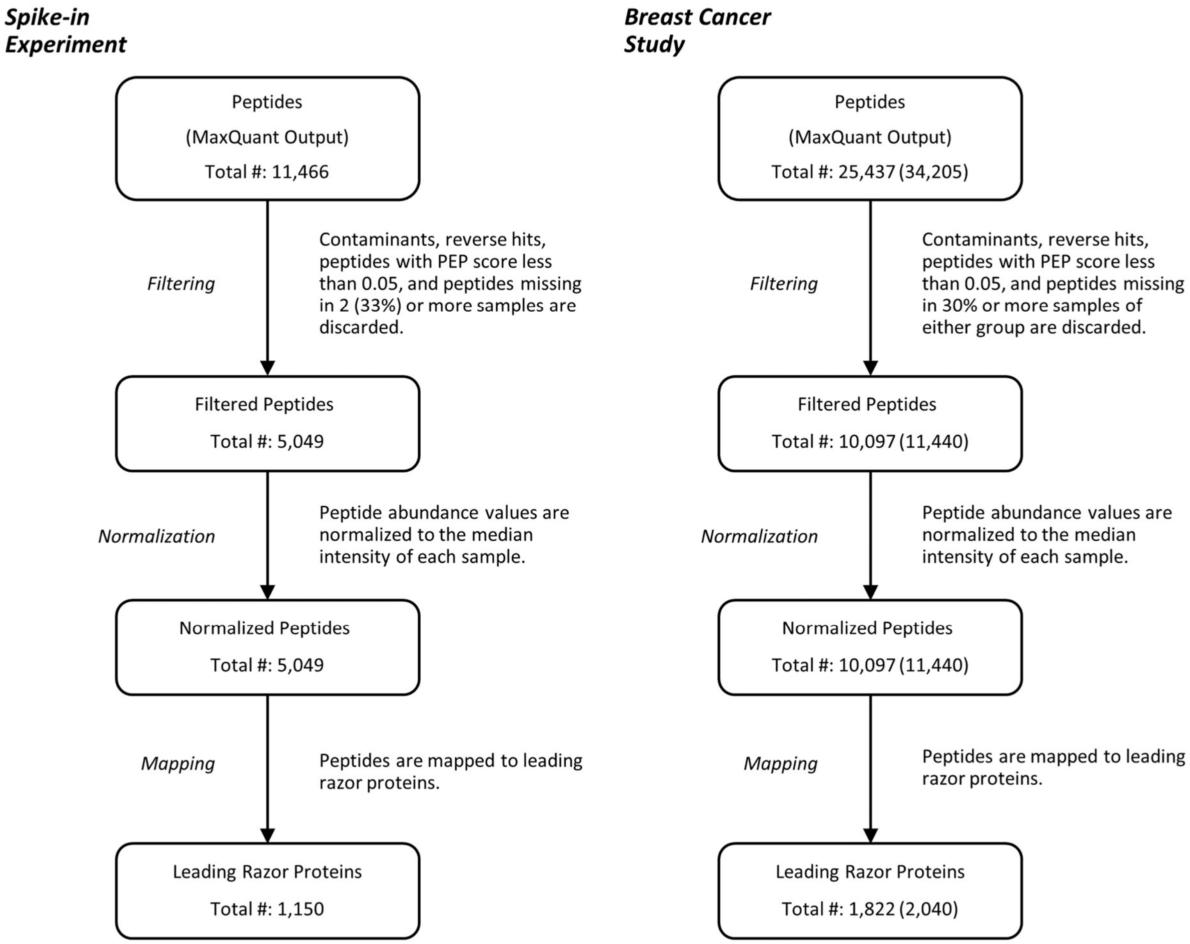

Supplementary Fig. 2. Steps of data pre-processing for the spike-in experiment and breast cancer study. The breast cancer study comprises two datasets: EMC and NKI-AVL + RUMC datasets[4]. Numbers outside (inside) the parentheses indicate the number of peptides/proteins retained at each stage in the EMC (NKI-AVL + RUMC) dataset. The filtering criteria were chosen to mimic the criteria specified in the original publications[4,5].

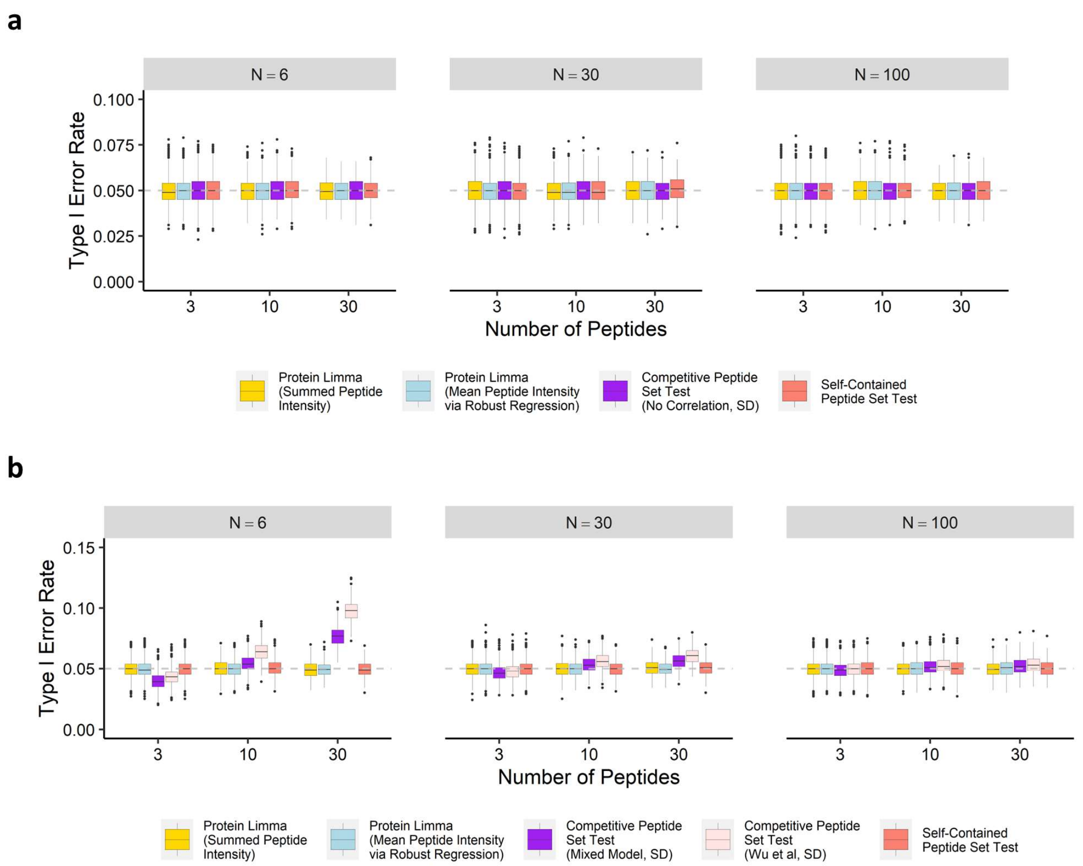

Supplementary Fig. 3. Type I error rates of different methods for differential protein expression analysis based on simulated data. Supplementary Table 2 provides detailed information about each method. The data were simulated with inter-peptide correlation coefficients (ρ) assumed to be **a** 0 or **b** 0.05. The simulated proteomes consisted of 3000 proteins with 3 peptides each, 900 proteins with 10 peptides each, or 300 proteins with 30 peptides each (with equal peptide distribution for all proteins). No proteins were assumed to be differentially expressed. The number of samples (N) was set to 6, 30, or 100. The simulation was repeated 1000 times.

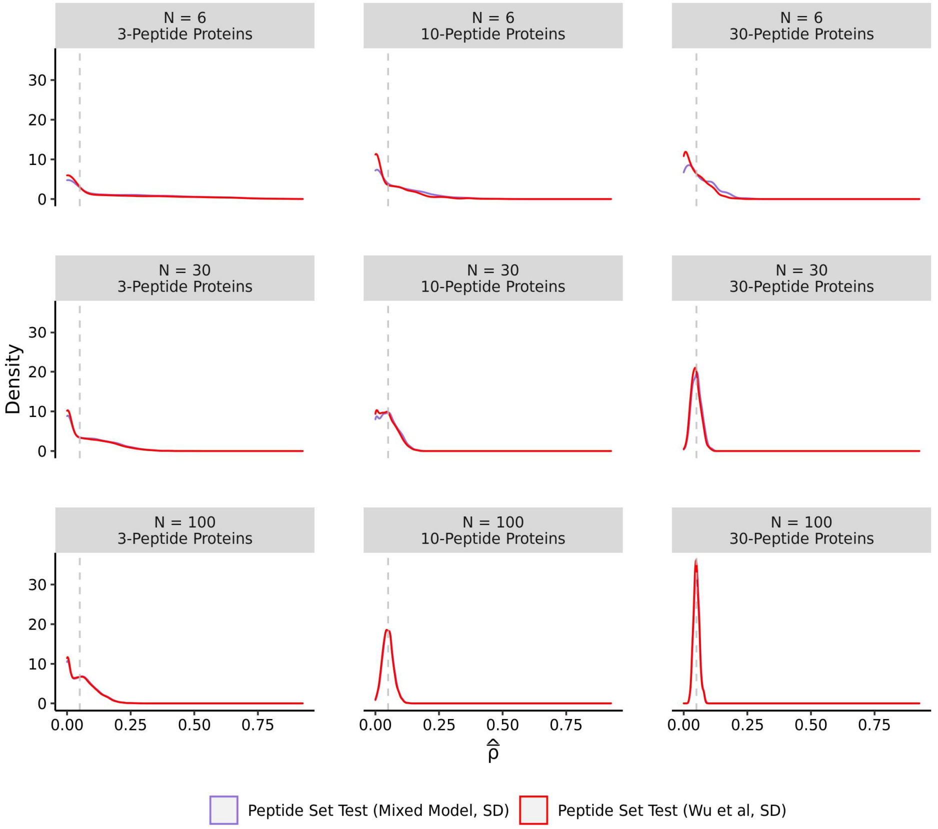

Supplementary Fig. 4. Comparison of inter-peptide correlation coefficient estimates. These density plots illustrate the distribution of inter-peptide correlation coefficients estimated by the mixed model approach and the original approach proposed by Wu et al[1]. The simulated proteome consisted of 3000 3-peptide proteins, 900 10-peptide proteins, or 300 30-peptide proteins. The inter-peptide correlation coefficient (ρ) was assumed equal to 0.05. The number of samples (N) was set to 6, 30, or 100. The vertical dashed lines represent the true ρ, which is equal to 0.05.

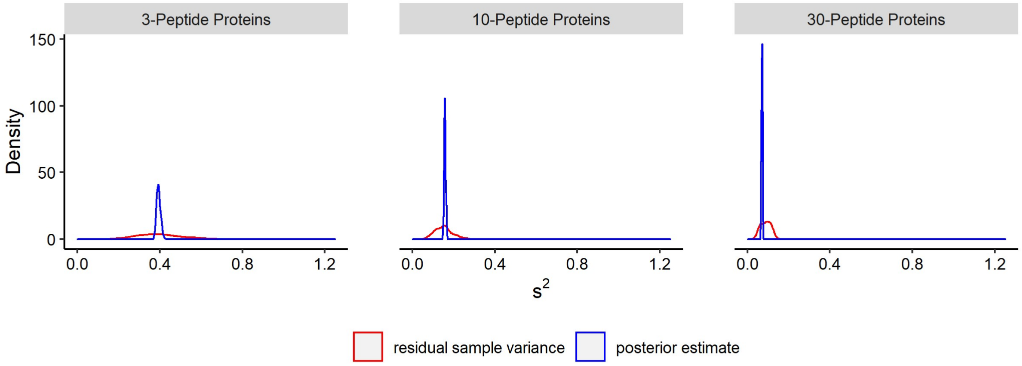

Supplementary Fig. 5. Comparison between residual sample variance and posterior estimates of sample variance. These density plots illustrate the distribution of residual sample variance and posterior estimates of sample variance for a simulated proteome comprising a mixture of proteins, including 1400 3-peptide proteins, 360 10-peptide proteins, and 40 30-peptide proteins. No proteins were assumed to be differentially expressed. The inter-peptide correlation coefficient (ρ) was assumed equal to 0. The number of samples (N) was set to 30. Estimates of sample variance were calculated by limma-trend[2]. Peptide abundance values were collapsed into protein abundance through robust regression of peptide intensities with M-estimation prior to performing a protein-level moderated t-test using limma[2].

a

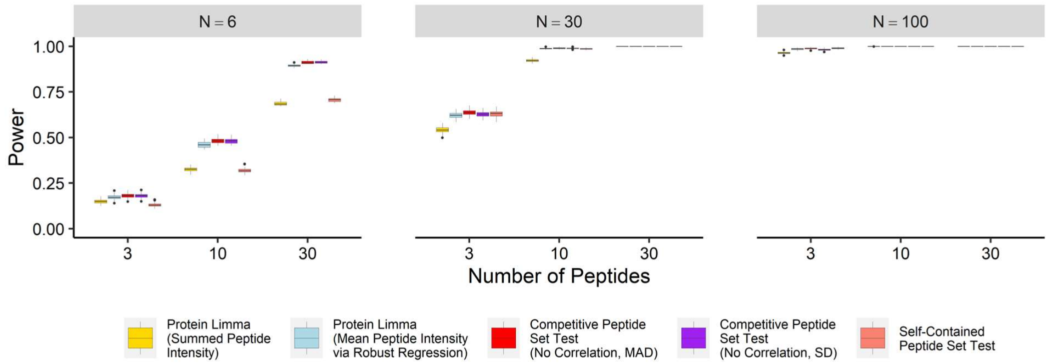

b

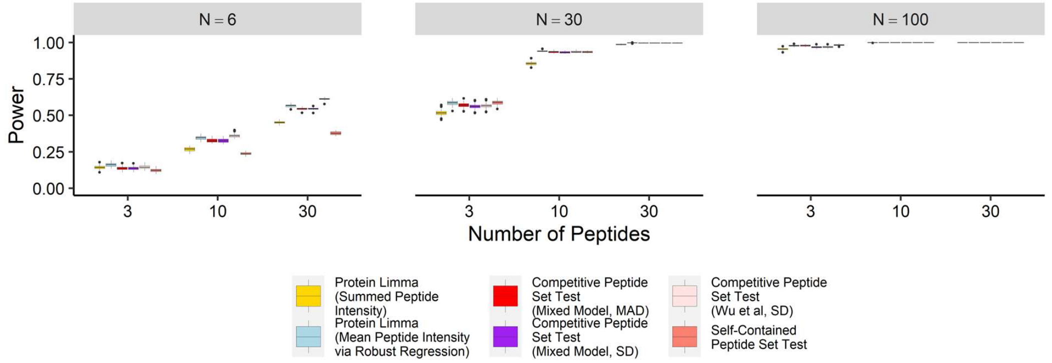

Supplementary Fig. 6. Power of different methods for differential protein expression analysis based on simulated data. Supplementary Table 2 provides detailed information about each method. The data were simulated with inter-peptide correlation coefficients (ρ) assumed to be **a** 0 or **b** 0.05. The simulated proteomes consisted of 3000 proteins with 3 peptides each, 900 proteins with 10 peptides each, or 300 proteins with 30 peptides each (with equal peptide distribution for all proteins). Five percent of proteins within each category were assumed to be differentially expressed. The number of samples (N) was set to 6, 30, or 100. The simulation was repeated 1000 times.

a

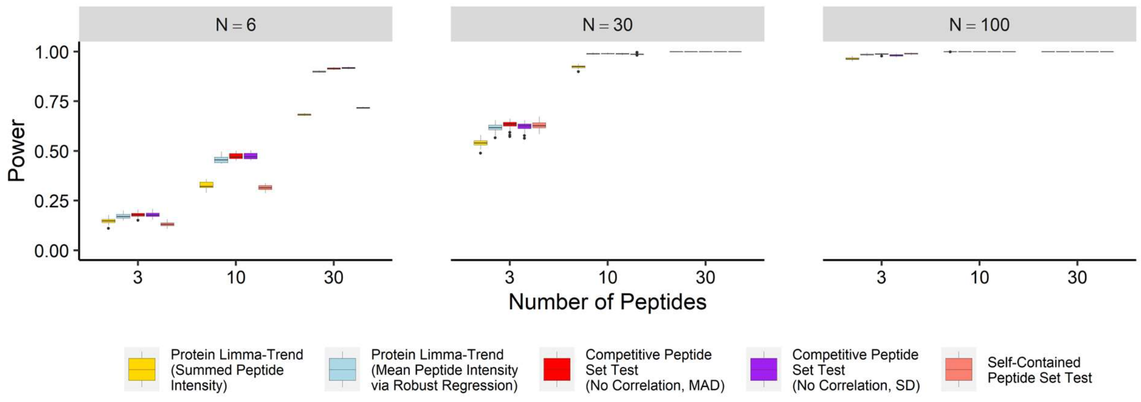

b

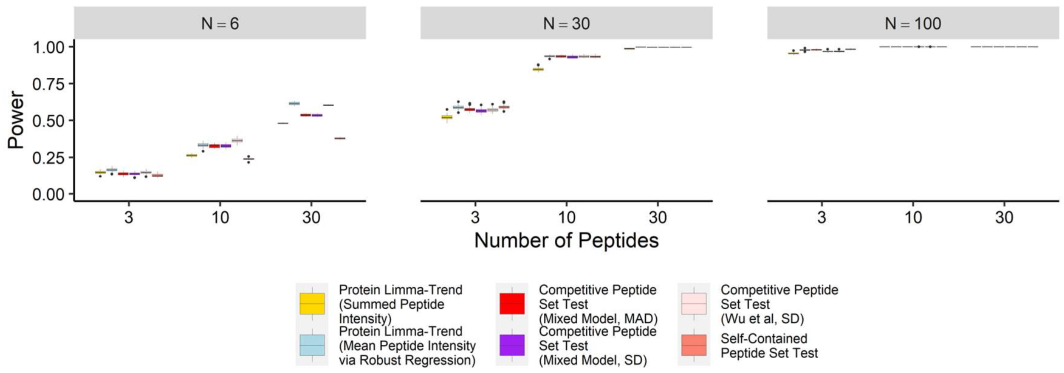

Supplementary Fig. 7. Power of different methods for differential protein expression analysis based on simulated data. Supplementary Table 2 provides detailed information about each method. The data were simulated with inter-peptide correlation coefficients (ρ) assumed to be **a** 0 or **b** 0.05. The simulated proteome contained a mixture of proteins, including 1400 3-peptide proteins, 360 10-peptide proteins, and 40 30-peptide proteins. Five percent of the proteins were assumed to be differentially expressed. The number of samples (N) was set equal to 6, 30, or 100. The simulation was repeated 1000 times.

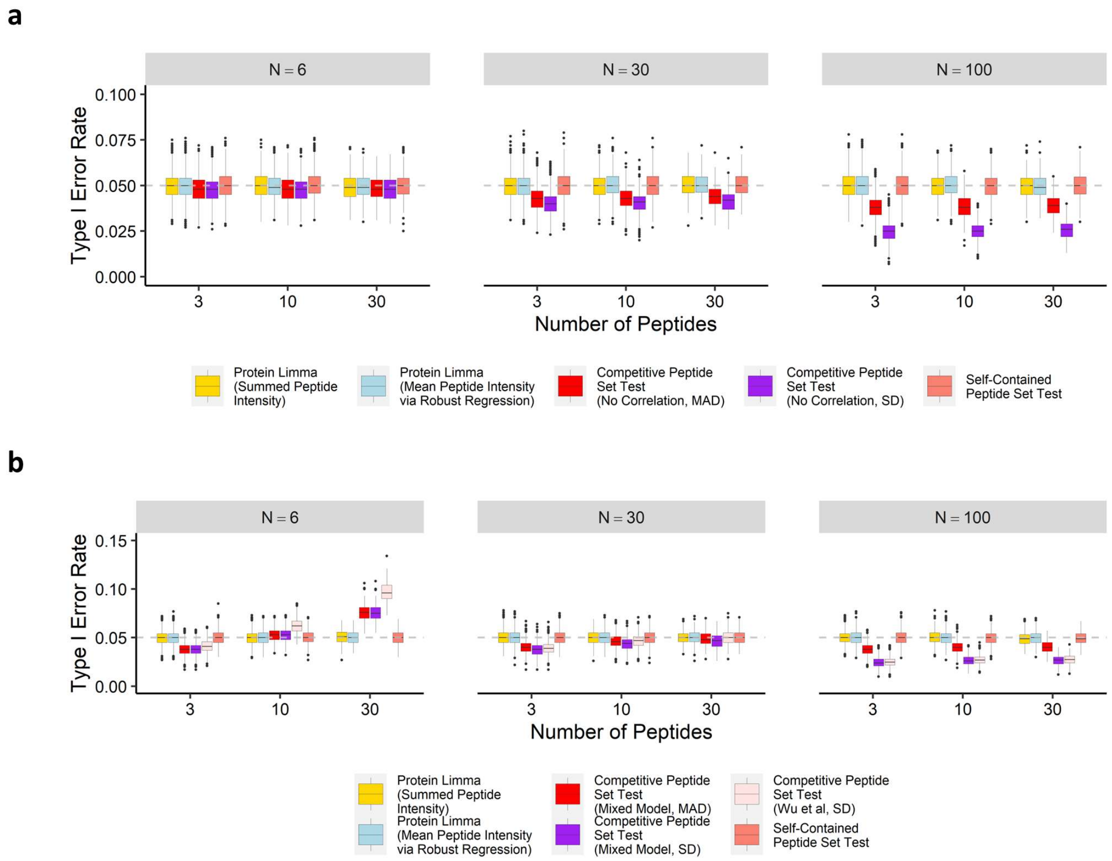

Supplementary Fig. 8. Type I error rates of different methods for differential protein expression analysis based on simulated data. Supplementary Table 2 provides detailed information about each method. The data were simulated with inter-peptide correlation coefficients (ρ) assumed to be **a** 0 or **b** 0.05. The simulated proteomes consisted of 3000 3-peptide proteins, 900 10-peptide proteins, or 300 30-peptide proteins (with equal peptide distribution for all proteins). Five percent of proteins were assumed to be differentially expressed. The number of samples (N) was set to 6, 30, or 100. The simulation was repeated 1000 times.

a

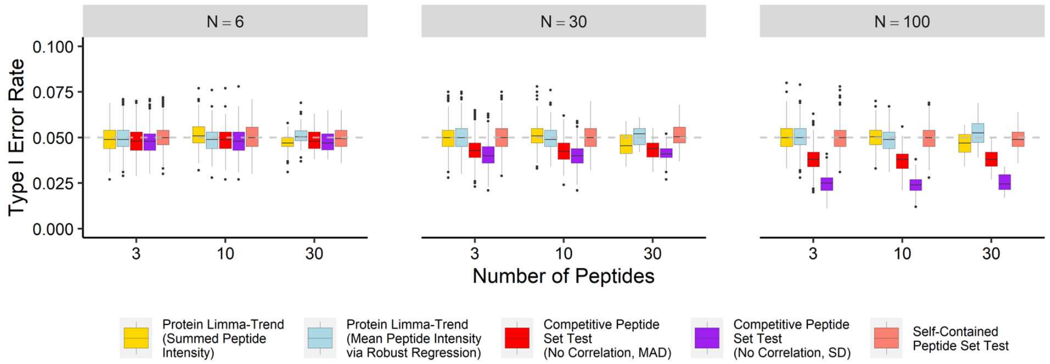

b

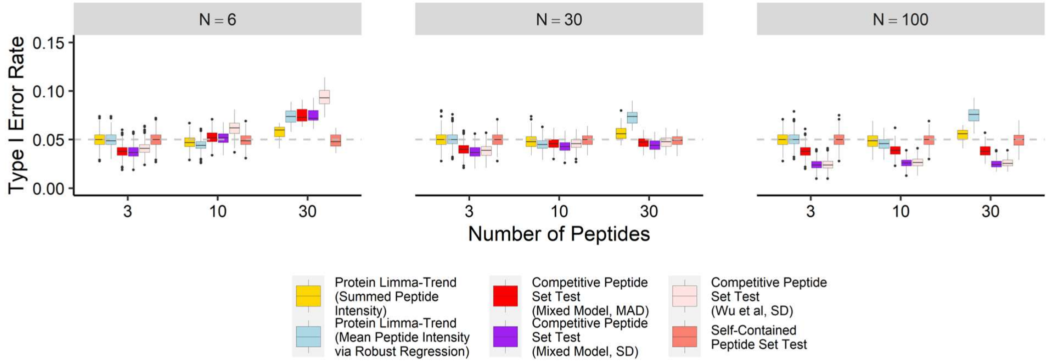

Supplementary Fig. 9. Type I error rates of different methods for differential protein expression analysis based on simulated data. Supplementary Table 2 provides detailed information about each method. The data were simulated with inter-peptide correlation coefficients (ρ) assumed to be **a** 0 or **b** 0.05. The simulated proteome consisted of a mixture of proteins, including 1400 3-peptide proteins, 360 10-peptide proteins, and 40 30-peptide proteins. Five percent of proteins were assumed to be differentially expressed. The number of samples (N) was set to 6, 30, or 100. The simulation was repeated 1000 times.

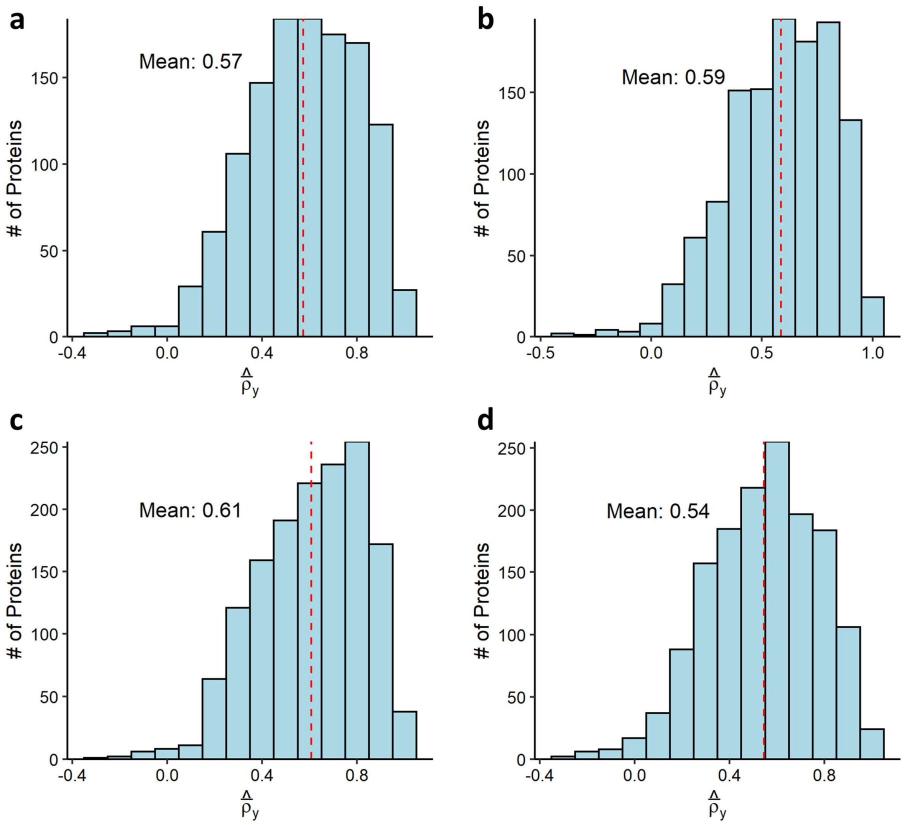

Supplementary Fig. 10. Averaged inter-peptide correlations for proteins calculated based on the breast cancer study. **a** Patients with good outcomes to tamoxifen treatment (i.e., hormone-sensitive) in the EMC cohort. **b** Patients with poor outcomes to tamoxifen treatment (i.e., hormone-resistant) in the EMC cohort. **c** Patients with good outcomes to tamoxifen treatment (i.e., hormone-sensitive) in the NKI-AVL + RUMC cohort. **d** Patients with poor outcomes to tamoxifen treatment (i.e., hormone-resistant) in the NKI-AVL + RUMC cohort.

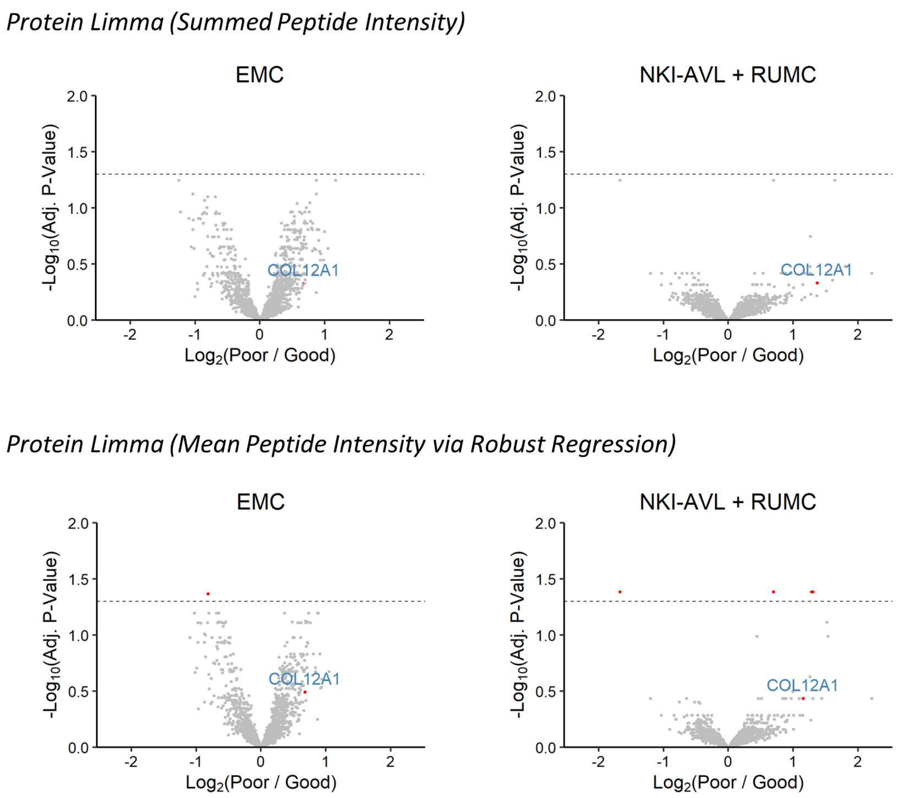

Supplementary Fig. 11. Volcano plots highlighting protein expression changes in tamoxifen-resistant breast cancer patients based on the results of aggregation-based approaches. Supplementary Table 2 provides detailed information about each method. Dotted horizontal lines indicate an adjusted p-value threshold of 0.05. Differentially regulated proteins are highlighted in red. COL12A1 is labeled.

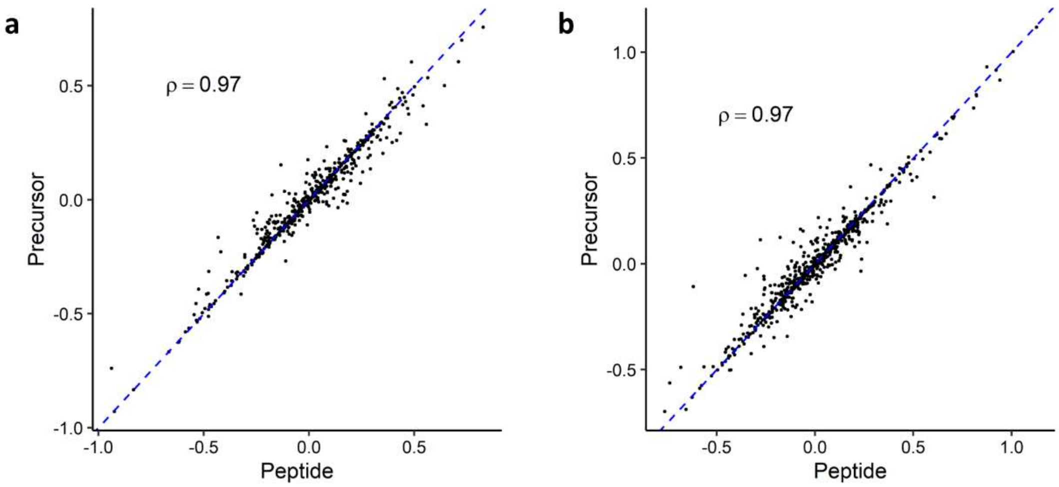

Supplementary Fig. 12. Evaluation of the correlation between $\log_2$ fold changes calculated using peptide-level data and precursor-level data. a EMC dataset. **b** NKI-AVL + RUMC dataset.

Supplementary References

1 Wu, D. & Smyth, G. K. Camera: a competitive gene set test accounting for inter-gene correlation. *Nucleic Acids Res* **40**, e133, doi:10.1093/nar/gks461 (2012).

2 Ritchie, M. E. *et al.* limma powers differential expression analyses for RNA-sequencing and microarray studies. *Nucleic Acids Res* **43**, e47, doi:10.1093/nar/gkv007 (2015).

3 Wu, D. *et al.* ROAST: rotation gene set tests for complex microarray experiments. *Bioinformatics* **26**, 2176-2182, doi:10.1093/bioinformatics/btq401 (2010).

4 De Marchi, T. *et al.* 4-protein signature predicting tamoxifen treatment outcome in recurrent breast cancer. *Mol Oncol* **10**, 24-39, doi:10.1016/j.molonc.2015.07.004 (2016).

5 Gatto, L. *R/Bioconductor for Mass Spectrometry and Proteomics*, <https://lgatto.github.io/2021-03-15-RProt-online/sec-quant.html> (2021).
